# Mechanism of Injury for Traumatic Mid-Foot Lisfranc Injuries: Impact of the COVID-19 Pandemic

**DOI:** 10.7759/cureus.58644

**Published:** 2024-04-20

**Authors:** Albert T Anastasio, Aman Chopra, Ryan M Ridenour, Chad E Cook, Amanda N Fletcher, Selene G Parekh

**Affiliations:** 1 Department of Orthopaedic Surgery, Duke University, Durham, USA; 2 School of Medicine, Georgetown University, Washington, DC, USA; 3 Department of Orthopaedic Surgery, Greater Pittsburgh Orthopaedic Associates, Pittsburgh, USA; 4 Department of Orthopaedic Surgery, OrthoCarolina, Charlotte, USA; 5 Department of Orthopaedic Surgery, Rothman Orthopaedic Institute, South Brunswick, USA

**Keywords:** case-series, mechanism of injury, mid-foot, covid-19, lisfranc

## Abstract

Background

During the COVID-19 pandemic, Americans transitioned away from their normal routines, drove in motor vehicles less, and reduced their physical activity, ultimately influencing the incidence and nature of orthopedic injuries that were operatively managed. The purpose of this study was to evaluate the effect of the COVID-19 pandemic lockdown and subsequent deconditioning on the mechanism of injury and severity of Lisfranc injury.

Methods

This retrospective study included patients with a traumatic Lisfranc injury who were surgically treated by a foot and ankle fellowship-trained orthopedic surgeon between 2015 and 2021. Electronic health records were queried for patient demographics, mechanism of injury, physical exam findings, and pain scores. Preoperative radiographs were reviewed to grade Lisfranc injuries using the previously described Nunley-Vertullo classification system. Descriptive and univariate statistics were performed to compare 15 patients in the pre-COVID-19 cohort and 15 patients in the post-COVID-19 cohort.

Results

In the pre-COVID-19 cohort, 80% (n=12/15) of the patients were female, the mean age was 46±15 years, the mean BMI was 29.7±7 kg/m^2^, and the mean follow-up period was 18.1±12 months. In the post-COVID-19 cohort, 53% (n=8/15) of the patients were female, the mean age was 48.5±17 years, the mean BMI was 31.4±7 kg/m^2^, and the mean follow-up period was 9.5±4 months. Significantly higher proportions of plantar ecchymosis (n=8/15, 53%), neuropathic pain (n=7/15, 47%), and swelling (n=12/15, 80%) were present in the post-COVID-19 cohort. A low-energy mechanism of injury was sustained by 73% (n=11/15) of the pre-COVID-19 cohort and 80% (n=12/15) of the post-COVID-19 cohort. Lisfranc injuries for the pre-COVID-19 cohort and the post-COVID-19 cohort demonstrated the following classifications: Grade 1 (33%, n=5/15 vs. 40%, n=6/15), Grade 2 (60%, n=9/15 vs. 53%, n=8/15), and Grade 3 (7%, n=1/15 vs. 7%, n=1/15).

Conclusion

Although a higher proportion of plantar ecchymosis, neuropathic pain, and swelling was observed, there was no association between a low mechanism of injury and a higher grade of Lisfranc injury following the COVID-19 pandemic.

## Introduction

In March 2020, the World Health Organization (WHO) announced the status of the COVID-19 virus as a global pandemic. Like much of the world, the United States (US) instituted widespread social distancing, shelter-in-place mandates, and quarantines to help reduce the spread of the virus. As Americans transitioned away from their normal day-to-day routines and commutes, less time in motor vehicles, and decreased participation in many physical activities were noted [1]. Thus, it was determined that the number of traumatic events and types of injuries being seen in trauma centers were changing [2].

Similar changes in traditional patterns of injury and disease onset were observed around the world and in many areas of medicine, including orthopaedics [3-9]. In Germany, there was an overall decrease in the absolute number of orthopaedic trauma cases, despite a rise in the proportion of traumatic injuries occurring within the household [10]. Comparable findings were found in India with respect to the total number of orthopaedic trauma cases decreasing, with a 25% increase in orthopaedic injuries related to a slip and fall mechanism at home and a decrease of about 45% in injuries related to motor vehicle accidents [11]. These findings are demonstrative in foot and ankle-related injuries. A 62% decrease in the total number of foot and ankle injuries was reported in a series from New York City, with a greater proportion of ankle fractures compared to ankle sprains, and a higher incidence of open fractures [12]. Whereas this data indicates that the total incidence of injury may be decreasing while injury severity is increasing post-pandemic, other data from professional sports leagues indicates that the occurrence of injury in sports may be increasing as a result of deconditioning and poor stress management response brought about by the pandemic [13-17].

Classically, Lisfranc injuries occur from high-energy mechanisms with a plantar flexed foot. Renninger et al. compared differences between low-energy and high-energy Lisfranc injuries and determined that low-energy injuries typically resulted in isolated ligamentous injuries that spared the lateral column [18]. No study to our knowledge has yet evaluated the effect of the COVID-19 pandemic lockdown and subsequent deconditioning on the mechanism of injury and severity of Lisfranc injury. Thus, the purpose of our study was to investigate mid-foot Lisfranc injuries during the COVID-19 pandemic. We hypothesized that a higher percentage of post-pandemic Lisfranc injuries would have occurred from lower-energy mechanisms and that lower-energy mechanisms would lead to injury patterns typically seen with higher-energy mechanisms.

## Materials and methods

Study design

This study is a retrospective review of consecutive patients who were surgically treated by a foot and ankle fellowship-trained orthopaedic surgeon at an academic institution. Institutional Review Board (IRB) approval was obtained prior to initiating the study. Utilizing current procedural terminology codes and procedural logs, patients were identified who underwent operative fixation for a traumatic Lisfranc injury (CPT code: 28585) between January 2015 and September 2021. Specifically, all patients not meeting exclusion criteria who underwent operative fixation between January 2015 and February 2020 were identified as the pre-COVID-19 cohort. All patients who underwent operative fixation between March 2020 and September 2021 were identified as the post-COVID-19 cohort. The month of March 2020 was chosen as the start-date cutoff of the post-COVID-19 cohort, as the first government-issued lockdowns in our state of practice occurred during this month. The pre-COVID-19 cohort date range was selected for the purpose of having a balanced number of patients in each cohort for analysis (15 total patients in each cohort). As a higher incidence of Lisfranc injuries occurred in our practice post-pandemic, the date range for the pre-COVID-19 cohort was substantially longer than the date range for the post-COVID-19 cohort. Patients under 18 years of age were excluded. Lastly, patients with follow-ups of less than six months were excluded from the study.

Patient variable data collection

Electronic health records were queried. For each patient, the following data was collected: mechanism of injury, demographics, body mass index (BMI), medical comorbidities, American Society for Anesthesiologists (ASA) physical status, and previous surgical history. Presenting symptoms and physical exam findings such as plantar ecchymosis, gross deformity, symptoms associated with neuropathic pain, and swelling were also recorded as present or absent. The final length of follow-up was determined from the time of Lisfranc surgery to the last clinic encounter. The numerical pain scores (scored from 0-10, with 0 being no pain, and 10 being maximal pain) were collected preoperatively and postoperatively. Postoperative complications and any reoperations such as removal of hardware were recorded.

Mechanism of injury

The mechanism of injury was classified as low-energy mechanisms versus high-energy mechanisms based on previously published studies [19,20]. Those mechanisms considered low-energy were athletic activity, ground-level twisting, or falls from less than four feet [19]. High-energy mechanisms of injury included falls from greater than four feet or direct crush injury [19,20]. 

Radiographic review and Lisfranc classification

Preoperative anteroposterior, lateral, and oblique radiographic images were obtained and reviewed. Utilizing the previously described Myerson and Nunley-Vertullo classification systems, each patient was assigned a grade or type of Lisfranc injury [19-21].

Statistical analysis

Descriptive and univariate statistics were performed. BMI, age, and follow-up periods were compared using Student's t-tests. Preoperative and postoperative pain score measurements were compared using paired t-tests. Proportions of comorbidities, ASA physical status >3, and injury signs and symptoms between pre-COVID-19 and post-COVID-19 cohorts were analyzed using Fisher’s exact two-sided test. Fisher’s exact analyses were used for nominal variables to evaluate differences between injury mechanisms. A Mann-Whitney U test was used to evaluate the differences between pre-COVID-19 and post-COVID-19 classification severity levels, which are ranked by three successively severe grades. A Mann-Whitney U test is appropriate for evaluating differences across ordinal categories in smaller sample sizes. Statistical significance was established at P< .05. All statistical analyses were performed using Microsoft Excel, version 16.52 (Microsoft Corporation, Redmond, Washington) and SAS® OnDemand for Academics (SAS Institute Inc., Cary, North Carolina).

## Results

Patient demographics

All patients with Lisfranc injuries (30 total) who met the inclusion criteria were included in this retrospective study; 15 patients were identified in the pre-COVID-19 cohort and 15 patients were identified in the post-COVID-19 cohort. In the pre-COVID-19 cohort, the majority of patients were female (12, 80%), the average patient age was 46±15 (range, 25-79) years, the mean BMI was 29.7±7 (range, 19-40) kg/m2, and the mean follow-up period was 18.1±12 (range, 6-43) months. In the post-COVID-19 cohort, the majority of patients were female (8, 53%), the average patient age was 48.5±17 (range, 21-82) years, the mean BMI was 31.4±7 (range, 21-50) kg/m2, and the mean follow-up period was 9.5±4 (range, 6-20) months. When compared, only the mean follow-up period was found to be significantly longer for the pre-COVID-19 cohort (P=.013) (Table 1).

**Table 1:** Lisfranc patient demographics and clinical data. Values in No. (%) or mean ± standard deviation (range). BMI: Body mass index, ASA: American Society for Anesthesiologists. Statistical analysis for patient demographics was performed using Student's t-tests and Fisher’s exact two-sided test.

	Pre-COVID-19 (n=15)	Post-COVID-19 (n=15)	P-value
Sex, female	12 (80%)	8 (53%)	
Age (years)	46.5±15 (25-79)	48.5±17 (21-82)	.74
BMI (kg/m^2^)	29.7±7 (19-40)	31.4±7 (21-50)	.52
Follow-up (months)	18.1±12 (6-43)	9.5±4 (6-20)	.01
Medical comorbidities			
Obesity	7 (47%)	5 (33%)	.71
Hypertension	5 (33%)	5 (33%)	1.00
Diabetes mellitus	1 (7%)	3 (20%)	.59
Active smoker	2 (13%)	2 (13%)	1.00
ASA physical status ≥ 3	1 (7%)	4 (27%)	.33
Injury signs and symptoms			
Plantar ecchymosis	0 (0%)	8 (53%)	< .01
Gross deformity	1 (7%)	1 (7%)	1.00
Neuropathic pain	0 (0%)	7 (47%)	< .01
Swelling	0 (0%)	12 (80%)	< .01
Removal of hardware	6 (40%)	2 (13%)	.21

Clinical data

Patient comorbidities and demographic factors are outlined in Table 1. Injury signs and symptoms of the pre-COVID-19 cohort included no (0%) patients with plantar ecchymosis, one (7%) patient with gross deformity of the midfoot, no (0%) patients with neuropathic pain, and no (0%) patients with swelling. Injury signs and symptoms of the post-COVID-19 cohort included eight (53%) patients with plantar ecchymosis, one (7%) patient with gross deformity of the midfoot, seven (47%) patients with neuropathic pain, and twelve (80%) patients with swelling. Significantly higher proportions of plantar ecchymosis, neuropathic pain, and swelling were present in the post-COVID cohort (Table 1).

Six (40%) patients in the pre-COVID-19 cohort and two (13%) patients in the post-COVID-19 cohort underwent removal of hardware eventually (Table 1). In the pre-COVID-19 cohort, a statistically significant improvement in numerical pain scores was observed when comparing preoperative and postoperative scores (4.7±2 vs. 2.2±2, P<.001). In the post-COVID-19 cohort, a statistically significant improvement in numerical pain scores was also observed when comparing preoperative and postoperative scores (6.1±2 vs. 4.3±4, P=.046) (Table 2). There were no reported complications following the procedure for either group.

**Table 2:** Numerical pain score for pre-COVID-19 and post-COVID-19 cohorts. Values in mean ± standard deviation (range). Pain score measurements were compared using paired t-tests. Preoperative to postoperative P-value indicates the P-value when comparing the change in preoperative pain score to postoperative pain score for both cohorts.

Comparison groups	Preoperative pain score	Preoperative P-value	Postoperative pain score	Postoperative P-value	Preoperative to postoperative P-value
Pre-COVID-19 (n=15)	4.7±2	.125	2.2±2	.06	< .01
Post-COVID-19 (n=15)	6.1±2		4.3±4		.04

Mechanism of injury

In the pre-COVID-19 cohort, 11 (73%) patients sustained a low-energy mechanism of injury. Of these 11, one (7%) patient sustained an injury during athletic activity, seven (47%) patients sustained a ground-level twisting injury, and three (20%) patients sustained a fall from less than four feet. Of the four (27%) patients who sustained a high-energy mechanism of injury, three (20%) patients sustained a fall from greater than four feet and one (7%) patient sustained a direct crush injury (Table 3).

In the post-COVID-19 cohort, twelve (80%) patients sustained a low-energy mechanism of injury. Of these twelve, three (20%) patients sustained an injury during athletic activity, four (27%) patients sustained a ground-level twisting injury, and five (33%) patients sustained a fall from less than four feet. All three (20%) patients who sustained a high-energy mechanism of injury experienced a direct crush injury. There were no significant differences in the proportions of low-energy and high-energy injury mechanisms and no significant difference between the Lisfranc injury classification between the two timeframes (Table 3).

**Table 3:** Lisfranc injury mechanisms and classification patterns. Values in No. (%). N/A: Not applicable. * Lisfranc injuries that could not be described with the Myerson classification system. Fisher’s exact test and Mann-Whitney U test were used to evaluate injury mechanisms and classifications.

	Pre-COVID-19 (n=15)	Post-COVID-19 (n=15)	P-value
Mechanism of injury			
Low energy			
Athletic activity	1 (7%)	3 (20%)	.59
Ground level twisting	7 (47%)	4 (27%)	.44
Fall from less than four feet	3 (20%)	5 (33%)	.68
High energy			
Fall from greater than four feet	3 (20%)	0 (0%)	.22
Direct crush	1 (7%)	3 (20%)	.59
Lisfranc injury pattern			
Nunley-Vertullo classification			
Grade 1	5 (33%)	6 (40%)	.36
Grade 2	9 (60%)	8 (53%)	
Grade 3	1 (7%)	1 (7%)	
Myerson classification			
Type B2	4 (27%)	4 (27%)	1.00
N/A*	11 (73%)	11 (73%)	

Classification of injuries

Using the Nunley-Vertullo classification system for the pre-COVID-19 cohort, five (33%) patterns were classified as Grade 1, nine (60%) patterns were classified as Grade 2, and one (7%) pattern was classified as Grade 3. In the post-COVID-19 cohort, six (40%) patterns were classified as Grade 1, eight (53%) patterns were classified as Grade 2, and one (7%) pattern was classified as Grade 3. Using the Myerson classification system, four (27%) patients were classified as sustaining Type B2 injuries in both cohorts. The remaining patients in both cohorts were not able to be classified using the Myerson classification as they did not have dislocation (Table 3).

## Discussion

The COVID-19 pandemic has shaped the landscape of orthopaedic foot and ankle trauma worldwide. An overall decrease in the total number of foot and ankle injuries with a higher incidence of more severe injuries has been reported [12]. In professional sports, particularly the National Football League (NFL), a higher incidence of injury has been reported post-pandemic [13-17]. No study has yet reported on the mechanism of injury and onset of Lisfranc injury in the wake of the COVID-19 pandemic. Thus, our hypothesis was that a higher percentage of post-pandemic Lisfranc injuries would have occurred from low-energy mechanisms and that low-energy mechanisms would lead to injury patterns typically seen with high-energy mechanisms. We determined that significantly higher proportions of plantar ecchymosis, neuropathic pain, and swelling were present on presentation in the post-COVID cohort (Table 1). Despite this finding, there were no significant differences in the proportions of low-energy and high-energy injury mechanisms and no significant difference in Lisfranc injury classification between the two timeframes. Furthermore, to reach an equal sample of 15 patients in the pre-COVID-19 group and 15 patients in the post-COVID-19 group, a date range roughly four times longer was required for the pre-COVID-19 group. Thus, at least in our specific patient population, the incidence of Lisfranc injury appears to have increased post-pandemic.

The finding of a higher frequency of Lisfranc injury in the post-pandemic period, while isolated to our specific practice, is not in concordance with the findings of other studies that have reported foot and ankle trauma incidents during the COVID-19 pandemic. Rydberg et al. report a 14% reduction in the average monthly rate of ankle fractures in 2020 when compared with monthly rates from 2017 to 2019 [22]. In the immediate 30-day period post-lockdown, a 26% decrease in fractures was seen [22]. Haskel et al. report an even sharper decrease of 77% in presentation for ankle fractures during the first month of the COVID-19 pandemic to a level 1 trauma center in New York City [23]. These results should be interpreted with caution, however, as the New York City lockdown was particularly strict, given the population density of the city and extreme hospital overcrowding resulting from the pandemic. Additionally, these authors report findings in the immediate aftermath of the lockdowns, whereas we report on a roughly one-and-a-half-year period after the first wave of lockdowns in our state of practice, North Carolina. Regardless, our finding of the increased frequency of Lisfranc injuries presenting to our clinic does contradict some of the existing data related to the incidence of lower extremity injuries in the wake of the COVID-19 pandemic.

In our cohort, while there were no significant differences in proportions of low-energy and high-energy injury mechanisms and no significant difference in Lisfranc injury classification, there were significantly higher proportions of plantar ecchymosis, neuropathic pain, and swelling observed on presentation in the post-COVID-19 cohort. Only one high-grade injury, as classified by the Nunley-Vertullo classification system, occurred in both the pre-COVID-19 group and the post-COVID-19 group. Thus, despite a priori power analysis, we may have been underpowered to detect differences in severity grading between the cohorts. It remains possible, therefore, that a higher frequency and severity of Lisfranc injury occur in the post-pandemic period. With regards to the mechanism of injury, three patients in the post-COVID-19 group presented with an injury sustained during an athletic event, while only one patient in the pre-COVID-19 group was injured during participation in athletics. This finding corroborates some of the existing data from professional sports leagues such as the NFL [13-17]. It has been postulated that alterations in preseason training programs may be responsible for the increased rate of injury seen in the post-pandemic period. The importance of preseason training programs in achieving improvements in lean body mass and preparing athletes for competition has been described [24]. Furthermore, rapid accelerations in workload can increase rate of injury in athletes [25]. Thus, with widespread reduction in physical activity across the US, the documented decreases in daily steps, the increase of sedentary behavior followed by the ending of lockdown and the return to both recreational and elite-level sports, deconditioning may contribute to the incidence of sport-related injuries seen in the post-pandemic period [1].

Our study does have several strengths compared to other studies reporting on the incidence of injuries after the COVID-19 pandemic. While many other studies have compared the post-pandemic period to only one year of the pre-pandemic period, we included patients over a five-year time frame, providing a more accurate representation of the years leading up to the COVID-19 pandemic. As a further strength of our study, all patients were seen by the same primary surgeon and clinical team, to allow for standardization of documentation of more subjective measures such as the presence of plantar ecchymosis and swelling. There are several limitations to our study as well. First, we only included patients presenting to one surgeon, limiting the generalizability of our findings to the population at large, where specific practice locations and patient demographics may substantially alter the mechanism of injury for Lisfranc injuries. Additionally, despite a priori power analysis, multiple trends were noted in the data which did not reach statistical significance, and the possibility of a type II error exists. However, with the low incidence of Lisfranc injury reported in the literature, our sample of 30 patients is relatively large for a single surgeon analysis [26].

## Conclusions

We observed a significantly higher proportion of plantar ecchymosis, neuropathic pain, and swelling on presentation for patients presenting with Lisfranc injury in the post-COVID-19 pandemic period. Athletic injuries were the causative factor for the Lisfranc injury in three patients in the post-COVID-19 group and one patient in the pre-COVID-19 group. Future research should aim to further describe injury patterns in the wake of the COVID-19 pandemic.
